# Field Demonstration of a Distributed Microsensor Network for Chemical Detection

**DOI:** 10.3390/s20185424

**Published:** 2020-09-22

**Authors:** Jeffrey S. Erickson, Brandy J. Johnson, Anthony P. Malanoski

**Affiliations:** Center for Bio/Molecular Science & Engineering, US Naval Research Laboratory, Washington, DC 20375, USA; brandy.white@nrl.navy.mil (B.J.J.); anthony.malanoski@nrl.navy.mil (A.P.M.)

**Keywords:** reflectance, portable, autonomous, sensor, porphyrin, color value, chemical detection, environmental monitoring

## Abstract

We have developed the ABEAM-15, a custom-built multiplexed reflectance device for the detection of vapor phase and aerosolized chemical plumes. The instrument incorporates fifteen individual sensing elements, has wireless communications, offers support for a battery pack, and is capable of both live and fully autonomous operation. Two housing options have been fabricated: a compact open housing for indoor use and a larger weather-sealed housing for outdoor use. Previously developed six-plex analysis algorithms are extended to 15-plex format and implemented on a laptop computer. We report the results of recent outdoor field trials with this instrument in Denver, CO in a stadium security scenario. Through software, the wireless modules on each instrument were configured to form a six-instrument, star-point topology, distributed microsensor network with live reporting and real-time data analysis. The network was tested with aerosols of methyl salicylate.

## 1. Introduction

Live and autonomous detection of chemical plumes is of importance for a number of applications such as force protection, air quality monitoring in industry, and threat detection or security at public events. In the past, large central testing laboratories have been utilized for this type of chemical detection. Samples are collected and sent away for analysis, a process that can take hours to weeks. Commercially available alternatives include devices like the handheld ion mobility spectrometer (IMS) or colorimetric test strips [1,2]. While these technologies can provide near real-time detection, they are not suitable for autonomous applications. Recently, the concept of distributed microsensor networks for chemical detection has become a topic of interest [3,4,5,6,7]. This strategy consists of sprinkling a large number of wirelessly networked devices across an area of interest. Although these devices typically offer lower sensitivity than gold standard assays, they have the advantage of potential proximity to the source of an event, resulting in exposure to higher concentrations of targets. The sensitivity tradeoff makes smaller, lighter, and lower power devices possible and helps to reduce per device costs.

A significant number of reports describe the development of array-based sensing approaches [8,9,10,11], including both electrochemical and optical approaches [8,9,12,13,14]. Optical methods range from image capture with post processing to simple color intensity measurements [9,10,15,16,17,18]. Commercial portable sensors for a range of applications have been described based on analysis of reflectance colors and/or intensities. Devices may be handheld or slightly smaller and utilize differential color measurements, often based on standards, for detection. The CZ-V20 series of RGB color sensors (Keyence America) is applied to the detection of packaging defects, for example. The CM-700D portable spectrophotometer (Konica Minolta), the Spectro Guide 45/0 (Premier ColorScan), the i-Lab (Photonics and Analytical Marketing), and a series of products by Ocean Insight, Inc. (formerly Ocean Optics, Inc.) provide full or partial reflectance spectra applicable to a number of scenarios. Further miniaturization of these measurements often uses an array of light sensors based on photodiodes with optical color filters. This may be as breakout boards (i.e., Parallax Color-PAL; Seeed Studio Grove I2C Color Sensor; Hamamatsu C9331) or stand-alone products.

**Figure 1:**
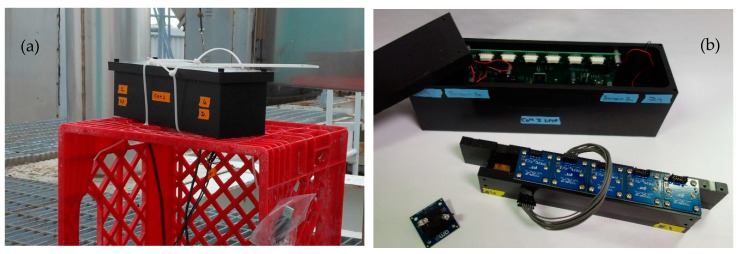
Images of the ABEAM-6 prototype sensor. The device includes six color-sensing breakout boards, a custom control board, fans, and wind tunnel indicator support in a custom housing. This device was used with external power and was controlled by a laptop computer. (**a**) The ABEAM-6 in an outdoor environment. (**b**) The partially disassembled ABEAM-6. Additional detail provided in the Supplementary Information, Figures S1 and S2.

Previously, we have reported the development of a six-indicator prototype reflectance sensor. The ABEAM-6 can be used in an open (i.e., indoor) format without housing or in a more ruggedized form for outdoor applications. The instrument operates in both live and autonomous modes, has been demonstrated in the laboratory [19] and outdoors [20], and has been utilized for the detection of a number of different chemical vapor targets, including alcohols [21], hydrogen peroxide [19], and a variety of toxic industrial chemicals (TICs) and traditional threat agents [22]. It has also been tested against aerosol targets [23]. For event identification, we have developed a data analysis algorithm that has been used as a standalone program for offline analysis [11,24] and as a component in our instrument control software GUI for real-time analysis during live data feeds. When in the housing, the instrument is 27.4 × 7.6 cm and 7.6 cm tall, weighs 1587 g, and consumes 300 mA of power at 7.5 V (Figure 1). As of 2015, the cost per unit was approximately USD 1100, excluding labor charges.

While the six-indicator prototype instrument has been used in a variety of demonstrations, it suffers from a number of drawbacks. The six-element array is sufficient for simple chemical discrimination in controlled environments, but higher multiplexing is desirable for real world applications. This instrument can be connected to a battery, but it has no onboard battery management electronics. In addition, this device has no wireless capabilities; only USB communications are available. Power and communications lines require penetration into the housing and make isolation of the internal circuit boards difficult. The impact of this fact was demonstrated during experiments with corrosive TICs; control board failures resulted from the corrosion of components [22]. Finally, the lack of wireless communications prevents the use of these devices in a distributed network. They could be deployed for such a use only by tethering each instrument to a laptop computer or other supplementary data logger with wireless communications.

## 2. Materials and Methods

**Figure 2:**
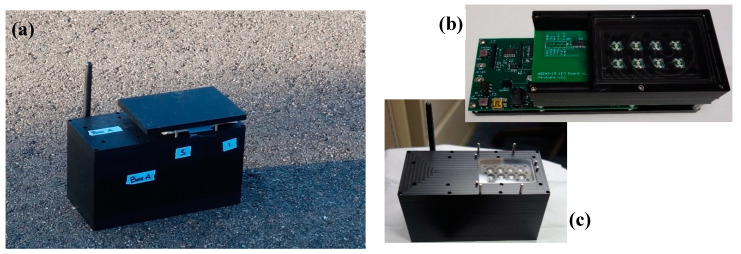
Images of the ABEAM-15 prototype sensor. The device includes fifteen surface-mounted RGB sensors, eight cool white LEDs, and three printed circuit boards. This device can be utilized autonomously or with a wireless tether to a laptop for real time detection. (**a**) The ABEAM-15 in an outdoor environment. (**b**) The ABEAM-15 board stack with indicator support. (**c**) The ABEAM-15 with sun cover removed. Additional detail provided in the Supplementary Information, Figures S3 and S4.

With lessons from the ABEAM-6 and a distributed network as the application in mind, this effort is currently working to develop a next-generation device, the ABEAM-15 (Figure 2). This device is intended to expand instrument capabilities while retaining similar size, weight, and power (SWaP) characteristics at a manageable cost. Three primary constraints were considered during this design process: the physical geometry of the instrument, the data sampling rate, and the autonomous deployment duration. In addition, the effort included two secondary goals: physical isolation of the instrument electronics from the environment and implementation of a compact indicator coupon on a single support allowing for deposition by a spot arrayer, inkjet printer, or similar piece of benchtop equipment. Finally, wireless communications and battery management were needed.

### 2.1. Device Geometry

The initial device design began with overall instrument geometry. The ABEAM-6 prototype utilized the commercially available TCS3200-DB color-sensing module from Parallax, Inc. as the detection element [11,20,21]. Each Parallax module is 3.43 × 3.43 cm in size and requires a 2.54 cm vertical standoff from the target for optimal color sensing. It includes a color-sensing integrated circuit (IC), two light emitting diodes (LEDs), and a lens. In the ABEAM-6, six of these Parallax modules are placed in single file orientation to form a wind tunnel geometry through which sampled air is pushed by fans. Each Parallax module uses a separate paper supported indicator bearing a single porphyrin or metalloporphyrin. Based on the size of the Parallax module, scaling this approach to support an increase in the number of indicator materials would result in a prohibitively large device. As an alternative strategy, the use of the packaged modules was abandoned and the color-sensing ICs were directly implemented as part of a custom circuit board.

The 2.0 mm × 2.4 mm flat no-lead (FN) package color sensors from AMS (TCS 34725) are significantly smaller than the Parallax modules, allowing more sensors to be packed into a small space; however, the sensors do not include hardware for focusing light. Incorporation of lenses required a shift from a single circuit board (a 2D design geometry) to a multi-layer board stack (a 3D design geometry). The three printed circuit boards were: a bottom control board, incorporating the microcontroller, data storage, USB and wireless communications, and battery management; a middle board with the 5 × 3 array of color-sensing ICs; and a top board with illumination components. Technically, the bottom and middle boards could be combined into a single layer. These components were separated to simplify changes to the supporting electronics without the need for the redesign of the optical train. Molded plastic aspheric lenses from Thorlabs (product # APL0609) were selected to keep the overall stack height short; these lenses are 6 mm in diameter and have a 9.07 mm focal length. The use of plastic lenses reduces the overall cost of the device, while the large numerical aperture of the aspheric design allows a significant fraction of scattered light to be collected. A custom plastic mount was designed to hold the 5 × 3 array of lenses between the middle and top layer boards. The diameter tolerance for the lenses was sufficient to allow a press-fit approach for mounting.

The illumination strategy for the reflectance sensor seeks to maximize the signal to noise ratio. In this implementation, incident light is provided at a 45-degree angle to the coupon. The color sensor is oriented normal to the surface of the coupon. Placing the lenses directly above the color sensors but below the LED board allows them to collect light from a large angle without affecting the illumination characteristics of the device. This strategy sets the overall geometry for the instrument and fixes the height of the different components. Because the color sensor is not an imaging detector, the lenses are not required to focus the incident light to a precise spot. This loosens the tolerance of the spacer heights considerably, allows the use of the press-fit approach for the lenses as opposed to a true optical mount, and opens up the possibility of using lightweight and inexpensive materials, such as plastics, as opposed to materials like aluminum.

Eight cool white LEDs in piranha packages (Product # R20WHT-F-0160, Bivar Inc., Irvine, CA, USA) were selected as the illumination sources based on geometric emission profile: the maximum intensity is at 45 degrees from normal. A plastic spacer above the top board provides holes designed to guide incident light at 45 degrees to the indicator elements while simultaneously rejecting reflected light from neighboring targets. A 50 × 75 mm glass microscope slide was mounted above this spacer and sealed with epoxy, providing a platform for indicator support. The epoxy seal completely separates the interior electronics from the environment, eliminating a major issue noted for the ABEAM-6 wind tunnel design. Alignment dowels on the top plastic spacer provide a guide for indicator coupon orientation. The alignment pins are staggered in an asymmetric pattern allowing for a single orientation and guaranteeing that spotted indicators are aligned with individual color sensors in the board stack. Note that in this design, exposure of the indicators occurs from the top, while detection occurs from the bottom. The indicator support material is paper, making it porous enough to allow permeation of targets throughout the material.

### 2.2. Sampling Rate

The expansion from six to fifteen sensing elements in the new design introduces a new problem, timing. In the ABEAM-6 prototype, each Parallax module was sampled sequentially. The heart of the Parallax module is the TCS 3200 color-sensing IC from AMS, which emits a pulse train proportional to light incident on its surface. It has three filters, giving four different responses: red, green, blue, and white. Each of these colors must be probed in sequence and integrated by the microcontroller. Approximately 200 ms is required to get a good dynamic range for each color (100 ms of bright time and 100 ms of dark time). For one complete sampling cycle, this totals to 4.8 s, 200 ms sampling time for each of the four colors over six total sensors [11,20,21]. The resulting sampling cycles for the ABEAM-6 were a fast 5 s cycle and a slower 30 s cycle. For the ABEAM-15, with fifteen sensing elements, multiple microcontrollers would be required to maintain a 5 s cycle. There is a secondary issue to be considered. The preference in a device of this type is that all indicators are interrogated at roughly the same time. In the ABEAM-6 design, successive sensors collect data points about 1 s apart. For a larger implementation, the total time required for a data cycle would be significantly longer, extending the timing differentials between the modules. In the ABEAM-15, the TCS 3200 color-sensing IC has been replaced with the newer TCS 34725. Here, the sensor provides the integration step and has an onboard analog-to-digital converter (ADC) that is interrogated by an embedded Atmel XMEGA 64A3U-AU microcontroller. Results for all four colors are reported by serial communication. This allows for initiation of all fifteen modules simultaneously, with simultaneous integration, and, finally, sequential reading of the data using the I2C protocol. While somewhat more complex, this approach allows for implementation of a 5 s sampling cycle with fifteen indicators.

### 2.3. Deployment Duration

The final variable considered was the desired autonomous deployment length. Deployment duration determines the required onboard data storage requirements and battery needs. For this design, a rechargeable battery, rather than a primary cell, was selected. US Navy regulations frown on the use of lithium battery chemistries, so nickel cells have been used. These batteries are heavier and larger than other alternatives. A 10 A-h nickel-metal hydride (NiMH) battery pack in a 6-S configuration was selected (Battery Space, Inc; #CU-JAS238). This battery pack is 17.8 cm long, 6.6 cm wide, 3.4 cm tall, and weighs approximately 1 kg. The length and width of the battery were used to guide the length and width of the circuit board stack (17.8 × 6.4 cm) and to define the profile of the outdoor housing described below. The battery capacity for this component should allow for at least one week of continuous operation when devices use a 30 s sampling cycle. Actual battery duration is difficult to address, because performance changes as batteries age and a series battery pack is limited by the lowest capacity cell in the chain. Based on the experimental performance of four devices, the utilized battery packs provide between 200 and 291 h of operational time at a 30 s sampling interval.

An AT25DF641 SPI serial flash memory IC (Adesto Technologies) was selected to provide 64 Mbits of long-term non-volatile data storage. Power interruptions will not result in the loss of data. The implemented firmware memory model provides storage for 97 h of data when a 5 s sampling cycle is used or 580 h of data on the slower 30 s sampling cycle. Power estimates, based on measurements from the final prototype device, suggest this battery and flash combination are appropriate. For a 5 s sampling cycle, the battery life is approximately 140 h, slightly longer than the memory capacity. For a 30 s sampling interval, the battery life is about 220 h, hitting the one week target duration. The longer operational duration at the 30 s sampling interval results from the less frequent use of the LED illumination sources, the highest current draw components in the device. The instrument active time is the same for the 5 s and 30 s sampling cycles, but there are six measurements by an instrument on a 5 s sampling cycle for every one by an instrument on a 30 s sampling cycle. A minimum of three battery changes would be necessary to fill the flash memory at the 30 s sampling rate.

The 6-S configuration of the battery pack provides a nominal output voltage of 7.2 V for the instrument. Each cell begins at approximately 1.5 V, has 1.2 V nominal power, and ends at approximately 1.0 V; the expected voltage of the pack varies from 9 V to 6 V. Battery management circuitry was introduced on the main control board. If the input voltage falls below 6.3 V, the microcontroller halts data collection, saves all data, and performs an orderly shutdown. If the instrument is connected to external DC power via the barrel jack, the battery will be disconnected and the instrument will utilize the DC source. This management strategy can be used to facilitate battery exchanges without the need to halt data collection. Instruments do not include internal battery charging mechanisms; batteries must be removed and recharged externally.

### 2.4. Device Housing

The instrument described in the previous section is partially sealed and can be used in mild environments; the devices have been used in this way for initial evaluations and screening of indicators. Both the USB plug and the DC power barrel jack are accessible, allowing for the possibility of unattended, continuous hardwired operation. In this format, the device is 17.8 cm long, 6.4 cm wide, and 8.9 cm tall including the battery pack. Total weight is approximately 1.3 kg (300 g without battery).

For more demanding environments, a protective housing is necessary (Figure 2). In designing this housing for autonomous use, access to the USB plug and the DC barrel jack were not prioritized; the battery chamber and wireless antenna were required. It was desirable to provide resistance to weather, and operation in the presence of ambient light is necessary. Because the new instrument does not include fans and does not use a wind tunnel geometry, the indicator coupons must be in contact with the air. At the same time, illumination must be controlled; ambient light is an insufficient light source for the device and fluctuations in illumination may impact results. Here, the housing provides a sealed shell. The design of this housing required minor revisions of the device: the top spacer and glass plate were replaced with larger ones. Disconnection of the wireless antenna is necessary for assembly. It may be necessary to externally seal the antenna penetration hole prior to deployment to prevent leaks. In this housing (excluding the antenna) the device is 19.1 cm long × 8.9 cm wide, and 11.7 cm tall (with sunshade in place). The device weighs approximately 2.2 kg (1.2 kg without batteries).

### 2.5. Firmware and Software

A detailed description of code development for the ABEAM-6 is available [7]. Briefly, firmware written in C controls sensors, accepts communications from a USB 2.0 connection, and serves as an XBee wireless endpoint. The employed Digi XBee Pro Series 3 wireless units provide an indoor range of 90 m and an outdoor range of 3.2 km. Management of the distributed network itself takes place in software responsible for controlling the wireless coordinator. In the star-point communications topology used, each sensor needs to communicate only with one module (the coordinator). As such, point to point wireless communications on the XBee modules could be implemented via transparent mode (AT mode) or using the more complicated API mode in firmware. For the ABEAM-15, the API mode implementation was used in firmware to avoid potential software issues. Data streams from the instrument contain 120 bytes of data per cycle. Transparent mode would require fragmenting the data into two or more packets, while API mode is capable of transmitting the data in one packet, provided there is no encryption or intermediate hops in between. Having all the data transmitted in one packet eliminates the following software issues: (1) every packet would be required to hold additional information about which portion of the data points it contains. (2) Lost data packets could cause failure of the dripfeed analysis, which requires the full cycle data in order to increment. (3) The software would be required to spend a significant amount of overhead verifying the transmission of all of the partial packets. For reference, API mode is required in software for all networks with multiple XBee endpoints.

Three elements are used to prevent packet loss in the wireless point-to-point communications network. First, transmission size is limited. The instrument sends data transmissions that are small, allowing each communication between firmware and software to be confined within a single packet. No fragmentation is needed, minimizing the effects of a packet loss. Second, error correction is employed. A checksum is generated at the point of transmission and reconstructed at the end of the transmission where it is checked for a match. An ack/nack signal is sent back to the instrument based on the results. Any transmissions with errors are re-sent. Third, basic communications strategies are used. Communications between the software and each instrument can be broken into two categories: expected responses and firmware-initiated communications. Expected responses are those where the software sends a command to an instrument and expects a response. In this case, determination of packet loss uses a timeout; if it is reached, the communication is re-tried. If there are repeat communication failures, the software determines that the instrument is offline. Firmware-initiated communication is more complicated; the software is not aware that a packet was sent. For the current implementation, the software simply stops communicating with the instrument, and it is considered to be offline.

Communication from the coordinator (computer) to each endpoint (instrument) is typically designed around single-letter ASCII commands. Communications from endpoint to coordinator are either ASCII commands or 120-byte payloads of data. Java and JavaFX were used to create a graphical user interface (GUI) for control of the instruments. The software organizes instrument operation into two separate working modes, live and autonomous. During an autonomous run, data are saved to the instrument flash. During a live run, data are transferred from the instrument to the software in real time. For autonomous mode, a single instrument is connected wirelessly or via USB, data collection is initiated, and the device is disconnected. For live mode, up to six instruments can be wirelessly connected to the controller in a star-point topology. In this way, distributed sensing is possible using either a group of autonomously running instruments with offline analysis after data collection, a group of wirelessly connected instruments providing data in real time, or as some combination of the two. Data analysis is a component of the software and is available for use either in real time or as an offline analysis utility.

### 2.6. Dripfeed and Data Analysis

The offline data analysis utility has been extensively described elsewhere [6,7,8] for devices with six indicators. Briefly, a threshold angle for event identification is fixed based on the first 120 data points (Background) collected by the device. Linear regression formulas are used to compute slope and r^2^ values for each color channel of each indicator (R, G, B for each indicator) for the most recent 20 data points and the Background window (next 120 most recent points). The cosine of the angle between the slopes is compared to that of the determined threshold angle for event identification. The algorithm can be varied to report an event based on changes at a single indicator or over multiple indicators. When an event begins, a window is opened during which any changes are considered part of the initial event; the length of this window can be adjusted, but typically 60 min is used. Additional details on the algorithm used are provided in the Supplementary Information (Figure S5).

Extension of this algorithm from six to fifteen indicators is straightforward. Real-time “dripfeed” analysis of live data feeds has been used for experiments with the ABEAM-6 [4]. In order to incorporate dripfeed analysis, the data analysis algorithm is inserted as a function or subroutine into the software code. Parameters such as the minimum number of indicators required for event reporting can be specified by the user before data collection is initiated. The instrument does require a warm-up period to initialize the algorithm (population of all data buffers). For a 5 s sampling cycle, 170 data points are necessary to fill the Background, Active, and Snap windows, requiring 15 min. For a 30 s cycle, 140 data points are required, collected over 70 min. Once initialization is complete, the device indicates a “negative” status and is ready for detection of an event.

**Figure 3:**
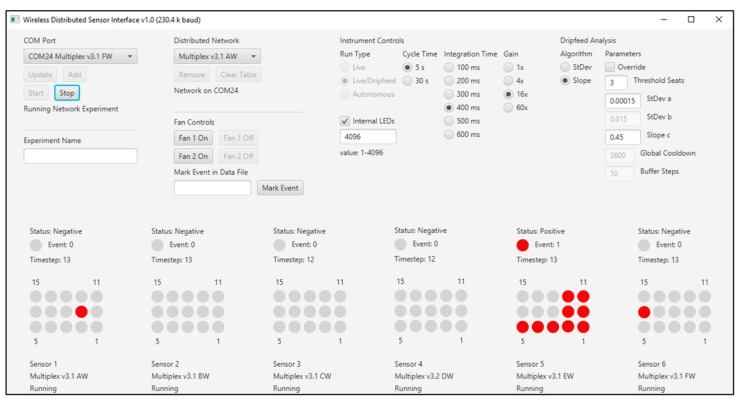
Image of the graphical user interface (GUI) for the ABEAM-15. This image shows single indicators under event detection conditions for Sensor 1 and Sensor 6, a condition insufficient for triggering positive status under the three indicator requirements being used. Sensor 5 is in positive status with nine indicators under event detection conditions. Here, a 5 s sampling cycle is used with 400 ms integration.

During live monitoring, each new data point is placed into the Snap and Active sliding windows, and all other points are shifted back one place. The oldest data point in the active window moves to the Background window, and the oldest data point in the Background window is dropped. Tests are performed for each indicator, and positive indicators are determined as previously described (above) [7]. A diagram of the fifteen indicators on the GUI provides information on positive indicators in red (Figure 3). This provides specific information to the user regarding which indicators have been triggered. If a sufficient number of positive indicators are observed, the status of the entire sensor will indicate “positive”. Other device status indicators are: “cooldown”, “extended”, and “OFF”. In addition to the graphical display of the current state of each sensor, log files are created and stored with the results of the analysis as it occurs.

## 3. Results

**Figure 4:**
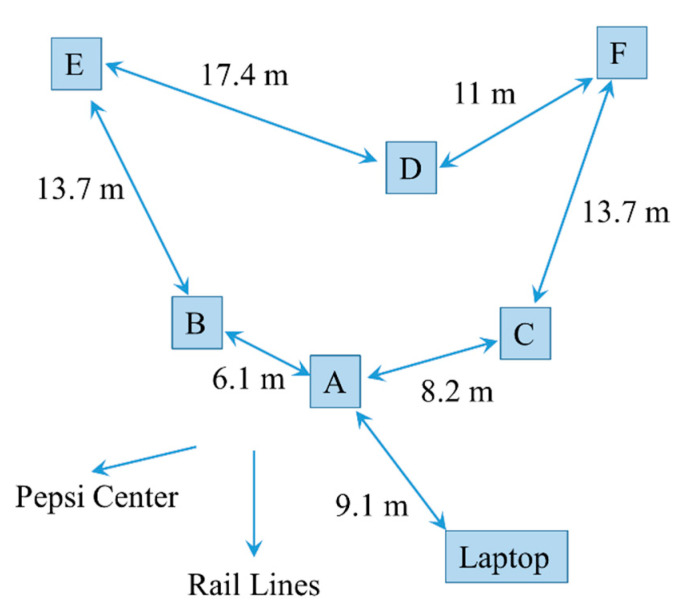
Distribution of devices during outdoor experiments.

A field demonstration of the ABEAM-15 technology was organized at the Pepsi Center in Denver, CO, USA on 4 October 2019. This is a facility providing between 17,000 and 21,000 seats (depending on configuration) for concerts, sporting events, and special events. Approximately 1 km from the central business district, it occupies 18 ha, including a number of parking lots, and is located in proximity to both commuter and freight rail lines. For this demonstration, six individual ABEAM-15 instruments in housings and two without housings were arranged in a parking lot located 480 m from the arena structure. The housed devices were arranged as shown in Figure 4, approximately 400 m from the rail lines.

**Figure 5:**
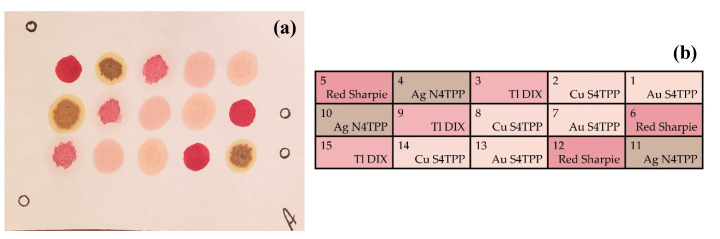
The coupon layout used for the trials described here. This coupon includes three copies of each of four indicators as well as three negative control spots created using red Sharpie^®®^ ink. Index markings at the edges of the coupon guide the orientation on the sensor device. (**a**) Photograph of a coupon. (**b**) Identification of the indicator locations used in the coupons.

All indicator coupons (Figure 5) incorporated three spots each of four metalloporphyrins, silver meso-tetra(4-aminophenyl) porphyrin (Ag N4TPP), thallium Deuteroporphyrin IX bis ethylene glycol (Tl DIX), and copper (Cu S4TPP) and gold (Au S4TPP) variants of meso-tetra(4-sulfophenyl) porphyrin, and three negative control spots were created using a red Sharpie^®^ marker. Coupons were prepared based on a modification of the previously used protocol [11,20] using Whatman filter paper as the support. Porphyrins were deposited by spotting 5 μL of the porphyrin solution (2 mg/mL) onto the appropriate area of the coupon. Samples were dried at 100 °C before storing in the dark.

Devices were initialized at 8:25 a.m.; it was 7.2 °C, sunny, and calm. All devices used 400 ms integration and a 30 s sampling interval. Following the initialization period, it became clear that something was causing variations in the data that were sufficient to set positive response conditions. Fluctuations in the light as clouds moved to block and unblock the sun were leading to these false responses. It was found that adding something near the boxes that could protect them from direct sunlight exposure (a sunshade) addressed this issue. The sensor at location C (Figure 4) lost communication with the wireless network at 10:38 a.m., just before exposures were initiated. As a result, those data have been excluded from the following discussion. The other five boxes collected 225 min of data.

**Figure 6:**
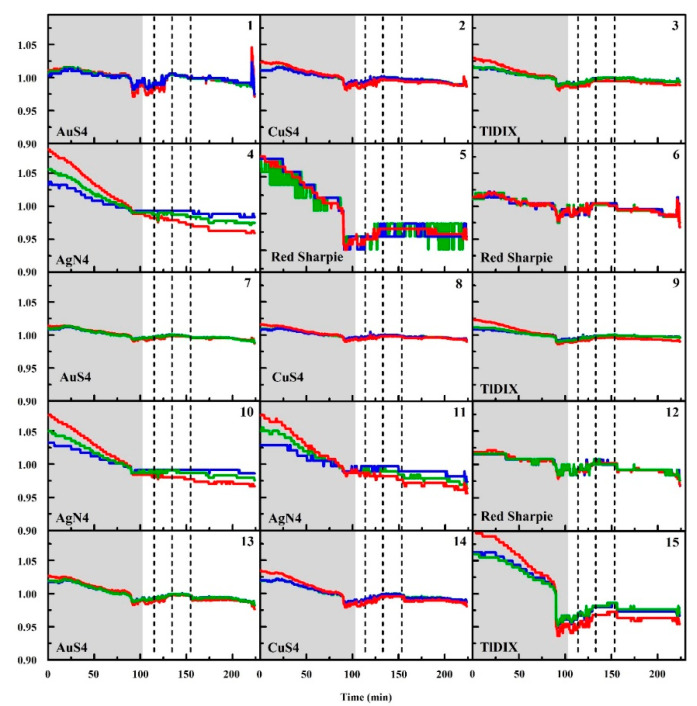
Normalized RGB data from the sensor located at position F (Figure 4). Dashed lines indicate exposure events; the gray shaded region is prior to installation of the sunshade. All three exposures resulted in positive responses from the device. Data are normalized to the average value of the color intensity over the duration of the experiment. Here, the color of the curve (red, blue, green) corresponds to the color value measured.

**Figure 7:**
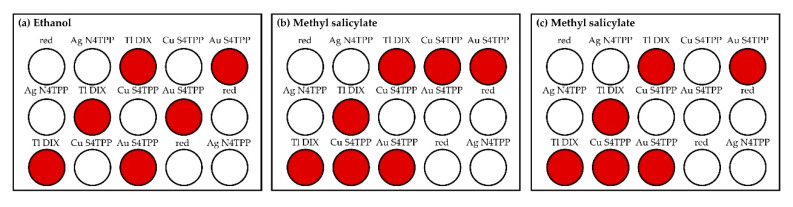
Indicator spot maps for the sensor located at position F (Figure 4) during exposure to ethanol (**a**) and methyl salicylate (**b**,**c**). Red circles are used to identify indicators that gave a positive response.

During the data collection period, exposures to ethanol and methyl salicylate were completed. The responses of all of the devices are summarized in Table 1. In addition to exposure responses, boxes A and B generated a positive response at 12:03 p.m. No other unprovoked responses were noted for any of the boxes after the sunshades were in place. Figure 6 provides representative data from the experiment. These data are from the device located at position F (Figure 4). For this device, the sunshade was placed at 09:54 a.m., triggering a positive response from all of the indicator spots in the array. At 10:19 a.m., the devices were exposed to an ethanol aerosol. All aerosols were generated using an atomizer spray bottle. The aerosol was delivered to each of the boxes individually from a distance of 1.5 m. The device at position F gave a positive response to this exposure, as shown in the spot map in Figure 7. This device also indicated an event upon exposure to methyl salicylate at 10:38 a.m.; the exposure was also an aerosol generated at a distance of 1.5 m (Figure 7). Finally, the device gave a positive response to methyl salicylate aerosol (11:00 a.m., Figure 7) generated at a point between boxes A and B.

## 4. Discussion

Here, we describe a new prototype sensor device utilizing a fifteen-element colorimetric detection array. This device is intended to provide chemical detection in a format suitable to long-term autonomous operation or as part of a wireless network. This prototype offers advantages over the previously described device [20] by expansion of the indicator array from six to fifteen elements. The higher multiplexing offers potential advantages in target discrimination and in the avoidance of false positives. This instrument also provides the capability for battery operation and wireless communication, features valuable in the use of the devices as a distributed network. Finally, the device housing seals the electronics, protecting them from environmental exposure as well as exposure to any corrosive chemicals.

This study provided an initial demonstration of the capabilities of these prototype devices. The coupons offered limited capacity with indicators selected based on a limited amount of data. Ethanol, a thoroughly studied target, was detected by all of the devices [11,20,21]. Mixed results were obtained for methyl salicylate, a target previously considered only as an interferent. Optimization of the array for the desired targets would be expected to provide improved performance and better discrimination of the compounds. The response of the devices to changing environmental illumination indicates the need for redesign of the housing. It is likely that this issue can be largely addressed by recessing the coupon slightly below the level of the surrounding surface. Together with the cover (Figure 2), this should provide the protection necessary while still allowing for exposure of the coupon without the need for fans.

Evaluation of the fifteen element prototypes in indoor and outdoor environments and work focused on improving those devices are ongoing. Additional target screening is focused on improving the libraries of responses for the porphyrin and metalloporphyrin indicators. Further expansion of the number of array elements could provide additional improvements to the capabilities of the device in both target discrimination and in allowing for the incorporation of additional indicator types. This type of modification will require redesign of the optical approach used in this device to add capacity while avoiding significant increases in size and weight. It should be possible to reduce the size of the spots and to adjust the spacing between them. Limitations to these dimensions will have to be determined and will require a change in the illumination approach. Additional indicator materials will also require reevaluation of the memory needed and likely expansion of that available on the current device.

## 5. Patents

Patent application: US 2017/0343471 30 November 2017 “Method for Analysis of Data Related to Use of Reflectance Based Color Changes in Real Time Sensing Applications,” A.P. Malanoski, B.J. White, J.S. Erickson, D.A. Stenger.

US 9,581,594 February 28, 2017 “Porphyrin-modified antimicrobial peptides for application as indicators of microbial targets,” C.R. Taitt, B.J. White.

## Figures and Tables

**Table 1:** Exposure response summary.

Time	Target	Device A	Device B	Device D	Device E	Device F
10:19	Ethanol	Positive	Positive	Positive	Positive	Positive
10:38	Methyl Salicylate	Positive		Positive	Positive	Positive
11:00	Methyl Salicylate	Positive				Positive

## Supplementary Materials

The following are available online at https://www.mdpi.com/1424-8220/20/18/5424/s1, Figure S1: ABEAM-6 block diagram; Figure S2: Photos of ABEAM-6; Figure S3: ABEAM-15 block diagram; Figure S4: Photos of the ABEAM-15; details on the detection algorithm utilized, Figure S5: Algorithm pseudocode.
