# Supplementary material for: Field Demonstration of a Distributed Microsensor Network for Chemical Detection
Source: Sensors (Basel). 2020 Sep 22;20(18):5424. doi: 10.3390/s20185424 (PMC7570817; doi:10.3390/s20185424)
Supplement: Supplementary file 1 [file sensors-20-05424-s001.pdf]

sensors

MDPI

Supplementary Information

Field Demonstration of a Distributed Microsensor Network for Chemical Detection

Jeffrey S. Erickson *, Brandy J. Johnson and Anthony P. Malanoski

Center for Bio/Molecular Science & Engineering, US Naval Research Laboratory, Washington, DC, USA 20375; brandy.white@nrl.navy.mil (B.J.W.); anthony.malanoski@nrl.navy.mil (A.P.M.)

* Correspondence: jeffrey.erickson@nrl.navy.mil; Tel.: +00-202-767-0364

Received: 28 August 2020; Accepted: 18 September 2020; Published: date

---

This supporting information provides additional detail on the prototype sensing devices including additional images and a block electronics diagram. Details on the detection algorithm are also provided.

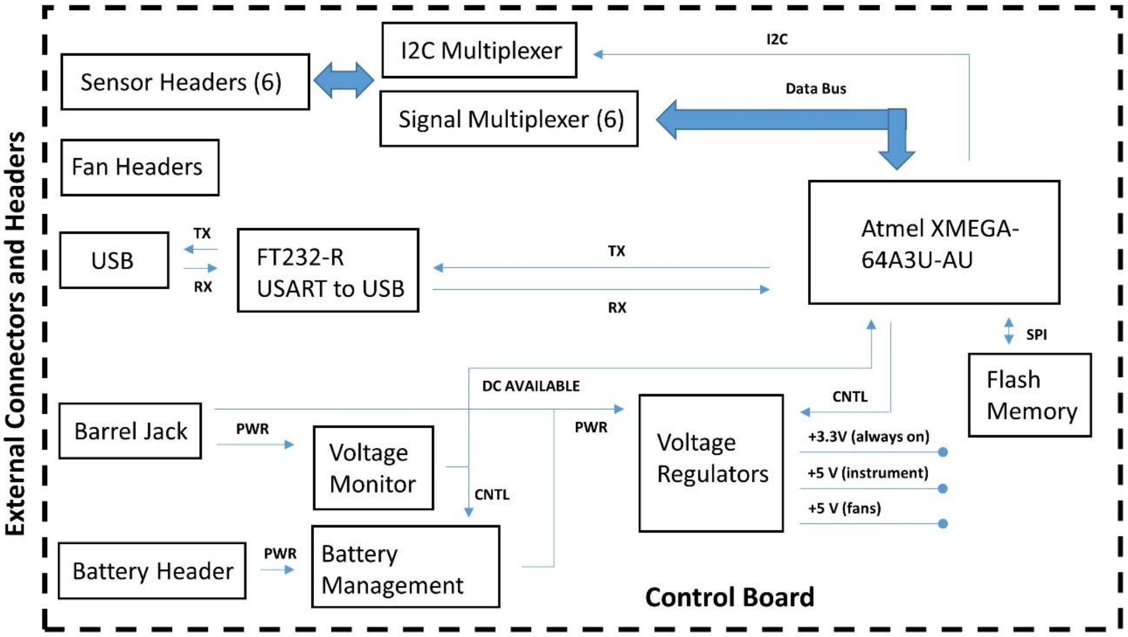

Figure S1. Block diagram for the ABEAM-6 prototype sensor.

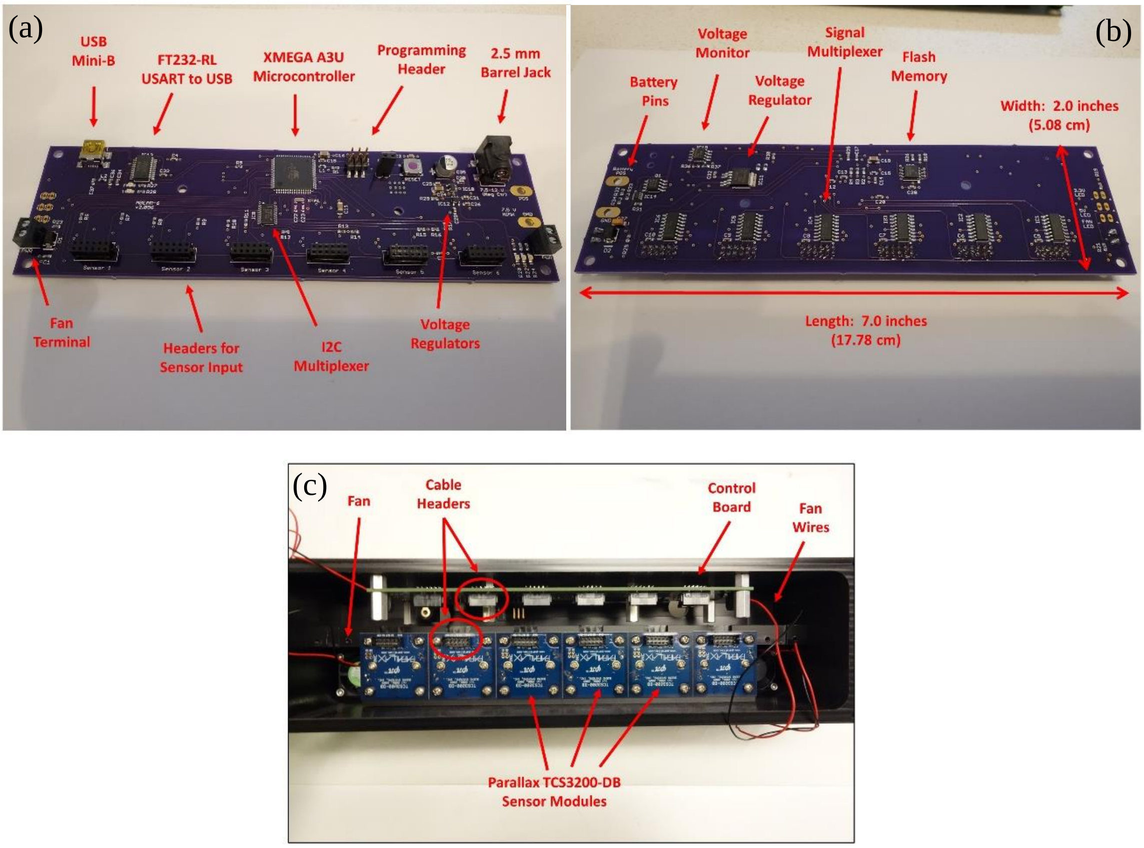

Figure S2. The ABEAM-6 control board is 7.0″ long x 2.0″ wide (17.78 cm x 5.08 cm). Selected components and dimensions have been labeled. (a) Front side. (b) Back side. (c) Control board mounted in a partially assembled housing (top view). Two fans and six Parallax TCS3200-DB sensors have been added. The cables that connect the sensors to the board have been removed for clarity.

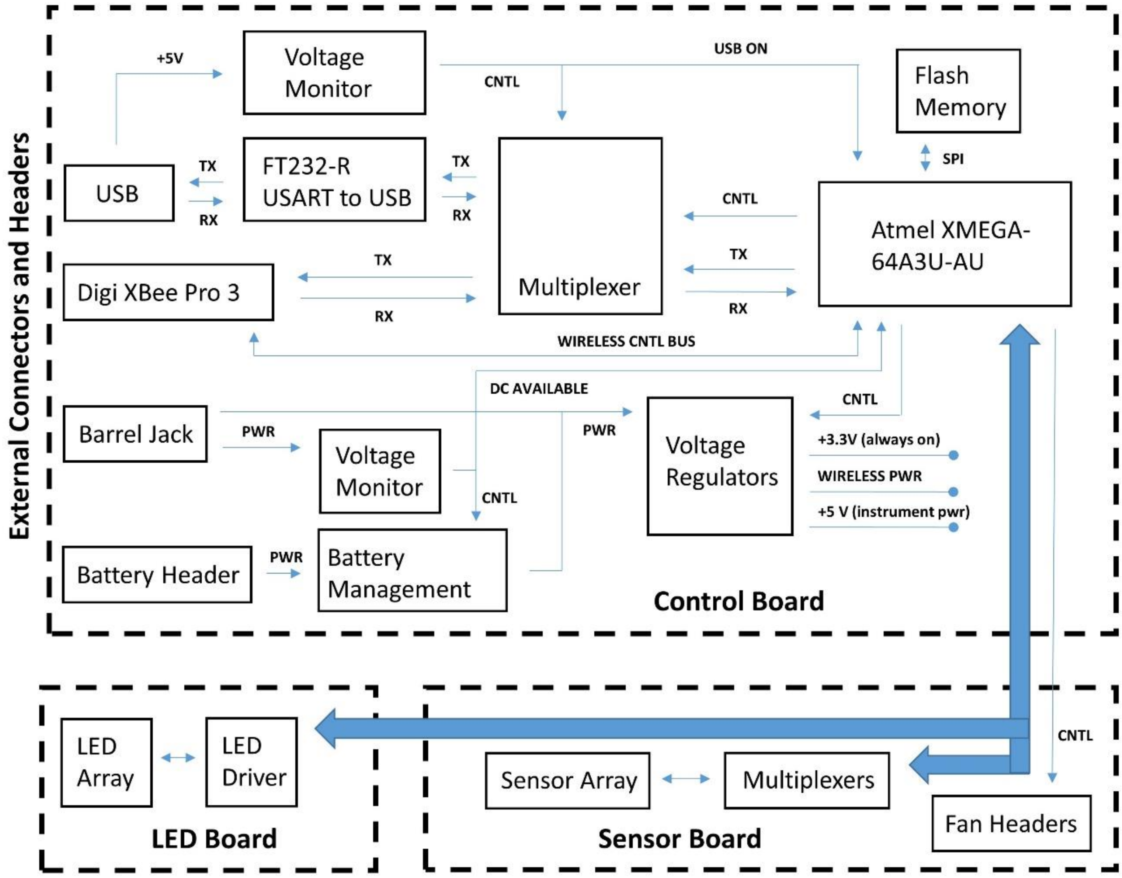

Figure S**3.** Block diagram for the ABEAM-6 prototype sensor.

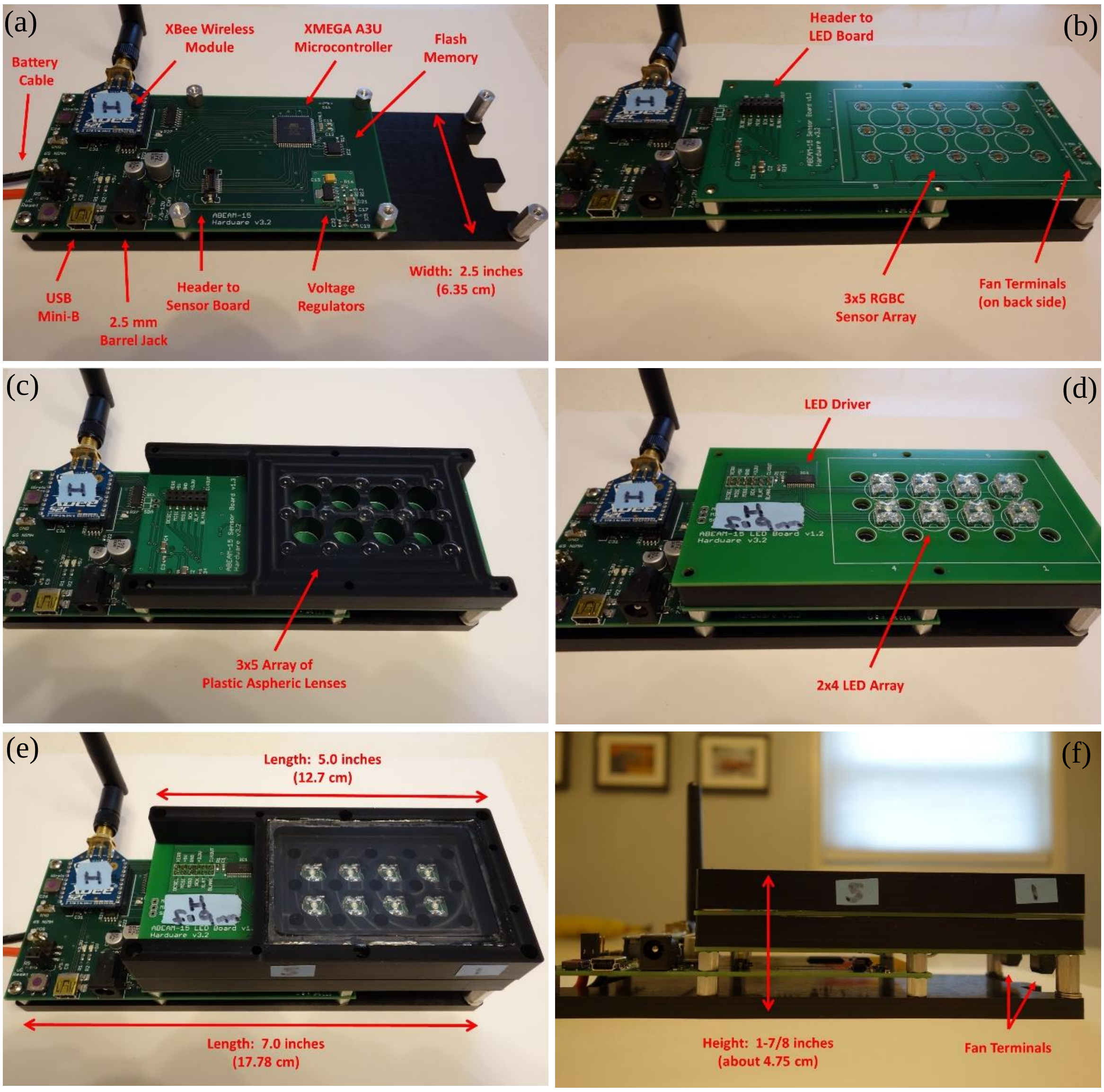

Figure S4. Top and side view photos of the ABEAM-15 circuit board stack in its indoor housing in various states of assembly. Photos of plastic mounts and spacers are included. All three circuit boards are 5″ long x 2.5″ wide (12.7 cm x 6.35 cm). Selected components and dimensions have been labeled. Note that some of the circuit boards have components on the back side; these are not shown. **(a)** Control board mounted to plastic base. The base is 7″ x 2.5″ (17.78 cm x 6.35 cm) and is designed to sit directly on top of the battery pack. **(b)** Sensor board added to the stack. **(c)** Lens mount added to the stack. Fifteen plastic aspheric lenses have been press-fit into the top of the mount. **(d** LED board added to the stack. The piranha package LEDs have an emission maximum between 30° and 50°. The ABEAM-15 is designed to illuminate the sample at a 45° angle. **(e)** Sample mount added to the stack. The optics are designed such that the sample should be placed directly on top of the glass plate. **(f)** Side view of the fully assembled stack. There are gaps between the boards in the photo because screws holding the stack together have not been completely tightened.

The Detection Algorithm.

The early sensor prototype had a reporting anomaly that lead to an intensity counter being prematurely dumped; the result is a low reported value for an individual color channel (R, G or B on a single sensor). These anomalous data values are excluded in the first round of signal processing by comparing each RGB channel value to that reported for the previous time point. If the absolute value of the difference between the two values divided by the previous value is greater than 35%, the previous value is substituted for the current value.

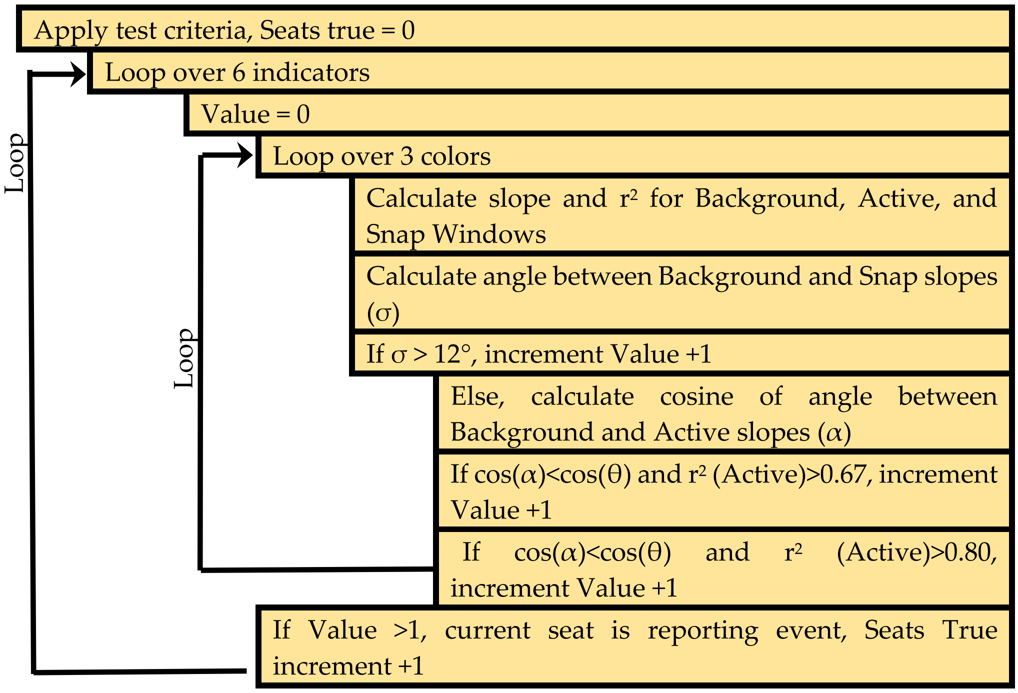

Figure S5. Psuedocode describing the sloped based algorithm.

Here, a slope based algorithm is used for event detection. The threshold angle for detection is fixed for each color value of each indicator based on the first 120 data points following initiation of the device. The value is determined using the following:

$$\emptyset = ae^{\frac{-RGB}{b}} + c \quad (1)\ (1)$$

Here, RGB is the initial intensity for a color channel. The parameters used depend on the minimum number of indicators required for detection (fixed by the user) or on the initial intensity values with a = 20 (one indicator, 70 for more than one indicator), and b = 130 (one indicator, 30 for more than one indicator), and c equal to the larger value of a user specified value (default 0.45°) or the angle of the dot product of the standard error calculated for the first 120 data points following initiation of the prototype.

Linear regression formulas are used to compute the slope and r^2 value for each color channel over two windows, Active and Baseline. The Active window comprises the 20 (30 s sampling interval; 10 min) most recent time points; the Baseline is a window that comprises the next 120 most recent time points. The cosine of the angle between the slopes for these windows is computed. If this value is less than the cosine of the threshold angle (Eq. 1) and the r^2 value for the current window is greater than 0.67 (one indicator, 0.57 for more than one indicator), the color value is counted as 1; if the r^2 value was greater than 0.8, the color is counted as 2. Counts from all color channels for a given indicator must sum to > 1 for the indicator to indicate event occurrence. High dose exposures can induce large or rapid changes; an additional criteria was included, using the 10 most recent data points. If the angle between the computed slope of this window (Snap) and the reference region slope is greater than 12° then the color contributes 1. After the tests have been applied to all indicators,

events are identified as changes in which the number of indicators reporting is greater than or equal to the minimum number specified (user defined). An event window was used for determination of the end point for detection. Here, that window is 60 min. Any indicator events within this window extended the event window by 60 min until no events are detected.

© 2020 by the authors. Submitted for possible open access publication under the terms and conditions of the Creative Commons Attribution (CC BY) license (http://creativecommons.org/licenses/by/4.0/).
